# Selenium Improves Physiological Parameters and Alleviates Oxidative Stress in Strawberry Seedlings under Low-Temperature Stress

**DOI:** 10.3390/ijms19071913

**Published:** 2018-06-29

**Authors:** Chongping Huang, Nannan Qin, Li Sun, Mingyan Yu, Weizhen Hu, Zhenyu Qi

**Affiliations:** 1 Department of Agronomy, College of Agriculture and Biotechnology, Zhejiang University, 866 Yu-Hang-Tang Road, Hangzhou 310058, China; 3120100028@zju.edu.cn (N.Q.); 21316135@zju.edu.cn (L.S.); 21516044@zju.edu.cn (M.Y.); 2 Agricultural Experiment Station of Zhejiang University, 866 Yu-Hang-Tang Road, Hangzhou 310058, China; zzjj@zju.edu.cn (W.H.); qizhenyu@zju.edu.cn (Z.Q.)

**Keywords:** strawberry, selenium, chilling stress, photosynthetic parameters, antioxidant enzymes, AsA-GSH cycle

## Abstract

Here, we investigated the effects of selenium (Se) applications on two strawberry varieties, Akihime and Benihoppe, under chilling stress and recovery conditions. Changes in photosynthetic parameters, antioxidant enzyme activities, ascorbate (AsA)-glutathione (GSH) cycle-related enzyme activities, and low-molecular-mass antioxidant contents were determined. Foliar spraying with Se alleviated the decline in the net photosynthetic rate and chlorophyll content and increased the malondialdehyde and hydrogen peroxide contents of strawberry seedlings’ leaves under chilling stress. As the time under chilling stress increased, the stomatal conductance decreased and intercellular CO_2_ concentration increased, suggesting that nonstomatal factors had major limiting effects on the net photosynthetic rate’s decrease. Se applications significantly alleviated the adverse impacts of chilling stress on changes in stomatal conductance and intercellular CO_2_ concentration. Se, especially at lower concentrations, significantly increased superoxide dismutase, catalase, and peroxide enzyme activities during chilling stress. Approximately 5 mg·L^−1^ of sodium selenite solution had the greatest stress-alleviating effects. Among the AsA-GSH cycle-related enzymes, ascorbate peroxidase, glutathione reductase, dehydroascorbate reductase, and monodehydroascorbate reductase (MDHAR) treatments, coupled with an appropriate dose of Se, significantly enhanced ascorbate peroxidase and MDHAR activities, which suggested that Se applications played important roles in strawberry leaves by affecting AsA-GSH cycle-related defenses against the oxidative damage caused by chilling stress. Furthermore, MDHAR was the key enzyme required to maintain the balance between AsA consumption and regeneration that may assist in protecting strawberry seedlings in a low-temperature environment.

## 1. Introduction

Strawberry (*Fragaria* × *ananassa* Duch.) is one of the largest fruit crops grown in the middle and lower Yangtze Regions during the winter season. It is popular with consumers because of its nutritional value and the limited availability of other locally produced fruit during the same season. Farmers grow strawberry crops in rotations with rice. Because the temperatures in the middle and lower Yangtze Regions during the winter and spring are quite temperate, ordinary farmers grow strawberries in greenhouses without heating capabilities. This can lead to short-term episodic (1 to 2 days) severe low-temperature injury. The optimum vegetative growth temperature for strawberries is ~20–30 °C, and lower temperatures limit leaf growth. Strawberry leaves do not suffer significant damage when the temperature decreases to −3 °C but are affected at −5 °C [1]. Low temperatures result in large losses in strawberry production, with severe frost damage resulting in a 20–30% reduction in yield owing to the damage to leaves and other organs [2]. It is therefore appropriate to undertake measures to alleviate the adverse effects of low temperatures on strawberry plants.

The physiological effects of selenium (Se) on humans and animals have been well documented. Se can enhance the activity levels of antioxidant enzymes in vivo and reduce oxidation. Studies have reported the physiological functions of Se during plant growth and development, as well as stress resistance, in a variety of crops [3]. Foliar spraying with an appropriate Se concentration can promote the early emergence and rapid development of rice tillers and increase the net photosynthetic rate (Pn) and chlorophyll content [4]. Se can protect sorghum leaves from high-temperature stress [5], wheat seedlings and *Dendrobium officinale* leaves from chilling stress [6,7], and olives (*Olea europaea* L.) from drought stress [8] by enhancing antioxidant defense systems. Se alleviates manganese toxicity by preventing oxidative stress in sunflower seedlings [9], and it inhibits cadmium accumulation and reduces lipid peroxidation in cucumber [10] and pepper plants [11]. Se can also regulate the subcellular distribution of antimony to reduce its toxicity in paddy rice [12].

Although strawberry is a winter crop, exposure to 5–0 °C results in chilling stress and serious physiological changes inside leaf cells [13]. This chilling stress causes the excess production of reactive oxygen species (ROS), such as superoxide radicals, hydroxyl radicals, and hydrogen peroxide (H_2_O_2_) [14]. To cope with the increased ROS levels, plants initiate a defense system to scavenge the ROS [3]. This system includes the antioxidant enzymes superoxide dismutase (SOD), catalase (CAT), and ascorbate peroxidase (APX), as well as several nonenzymatic antioxidants, such as reduced ascorbate (AsA) and glutathione (GSH) [15]. In some cases, the AsA-GSH cycle is considered important for plant defenses against stress-induced oxidative damage [16,17]. There is, however, little information available regarding the effects of Se on antioxidant enzymes and nonenzymatic antioxidants in strawberry crops in response to stress.

The aim of this study was to elucidate the role of Se in the regulation of photosynthetic parameters, antioxidant enzyme activity levels, and the AsA-GSH cycle in strawberry leaves under low-temperature stress to provide further insights into Se-mediated antioxidant metabolism under chilling stress and potential management measures.

## 2. Results and Analysis

### 2.1. The Photosynthetic Parameters Were Significantly Affected by Se Applications 

**Figure 1:**
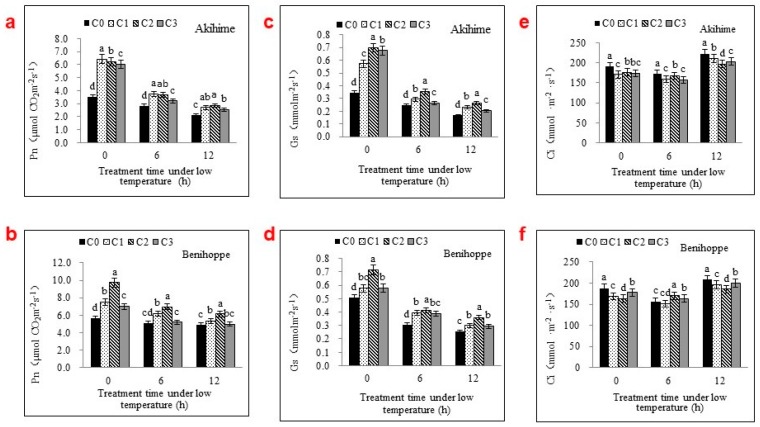
Effects of different exogenous Se treatments (C0, 0 mg·L^−1^; C1, 2.5 mg·L^−1^; C2, 5 mg·L^−1^; C3, 10 mg·L^−1^) on net photosynthetic rate (Pn) (**a**,**b**), stomatal conductance (Gs) (**c**,**d**) and intercellular CO_2_ concentration (Ci) (**e**,**f**) in leaves of two strawberry varieties under chilling stress. Bars with different letters are significantly different at the 0.05 level (LSD test).

As shown in Figure 1a,b, the exposure of strawberry plants to chilling stress decreased the Pn. However, this effect could be removed by applying exogenous Se. Seedling leaves receiving different concentrations, C1, C2, and C3, of exogenous Se demonstrated significant increases in the Pn of 66.07%, 102.60%, and 96.18%, respectively, for Akihime, and 13.90%, 52.70%, and 6.42%, respectively, for Benihoppe, at the beginning of chilling stress (0 h) when compared with C0. With extended low-temperature exposure, the Pn decreased gradually and the application of Se retarded this decline. After 6 h of chilling stress, the Pn values of Akihime leaves in treatments C1 and C2 were significantly greater than that of the control group, increasing by 32.93% and 30.87%, respectively. After the 12-h treatment, C1 and C2 increased by 28.32% and 35.27%, respectively, compared with C0. The Pn increase in C3 was significantly less than those in C1 and C2. Similar changes were found in Benihoppe.

As shown in Figure 1c,d, the Gs decreased as the exposure time to low temperature increased from 0 to 12 h. Foliar sprays containing different Se concentrations could alleviate the decline of Gs in strawberry seedlings’ leaves under chilling stress. Among the treatments, C2 (5 mg·L^−1^ Se) produced a greater Gs value and had more protective effects on chilling-injured plants.

As shown in Figure 1e,f, Ci increased as the exposure time to low temperature increased from 0 to 12 h. Exposure to exogenous Se resulted in a significant decrease in Ci values compared with the control. For Akihime treated at 0 °C for 6 h, the Ci values of exogenous Se-treated (C1, C2, and C3) strawberry leaves decreased by 8.40%, 13.77%, and 8.03%, respectively, when compared with C0. As the treatment time extended to 12 h, the leaf Ci values increased further. Applications of exogenous Se had significant stimulatory effects on Ci values. The C1 and C2 concentrations had greater effects on both strawberry varieties than the C3 concentration.

### 2.2. The Effects of Se Applications on Chlorophyll, MDA, and H_2_O_2_ Contents

**Figure 2:**
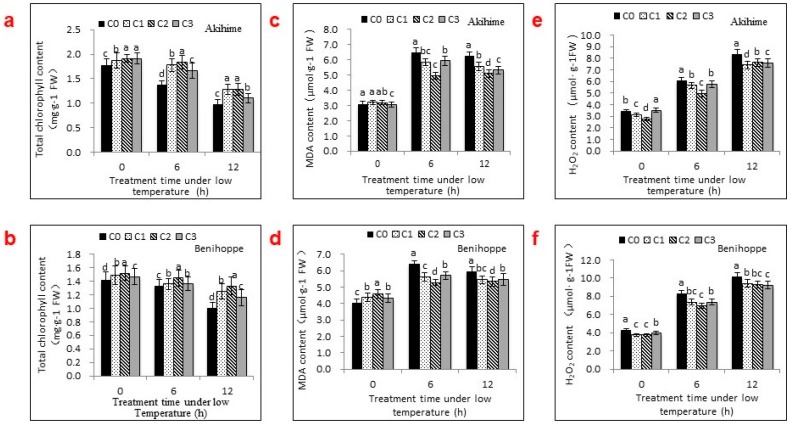
Effects of foliar spraying solutions containing different Se concentrations (C0, 0 mg·L^−1^; C1, 2.5 mg·L^−1^; C2, 5 mg·L^−1^; C3, 10 mg·L^−1^) on chlorophyll (**a**,**b**), MDA (**c**,**d**), and H_2_O_2_ (**e**,**f**) contents in the leaves of two strawberry varieties under chilling stress. Bars with different letters are significantly different at the 0.05 level (LSD test).

Foliar sprays containing different Se concentrations could alleviate the decline in the chlorophyll contents in strawberry seedlings’ leaves under low-temperature stress (Figure 2a,b). Plants receiving treatments C1, C2, and C3 had higher chlorophyll contents than C0-treated plants, regardless of the chilling stress duration (0, 6, or 12 h). For Akihime, the 6-h treatment at 0 °C increased the leaf chlorophyll content by 30.27%, 34.81%, and 21.17%, respectively, when compared with C0. For Benihoppe, the effects of exogenous Se on the leaf chlorophyll content increased significantly relative to the 6-h treatment, particularly with the C1 and C2 concentrations. Among the treatments, C2 produced a greater chlorophyll content and had more protective effects when used on chilling-injured plants.

The MDA content was measured to verify the hypothesis that Se applications could alleviate oxidative stress caused by chilling. The MDA content decreased significantly with all treatments (Figure 2c,d), from C1 to C3, before increasing as the Se concentration increased. C2 produced the greatest effects.

Several stress conditions cause the excessive accumulation of H_2_O_2_ and the production of other ROS that induce membrane damage. In both strawberry varieties studied here, the H_2_O_2_ content increased significantly as the exposure to low-temperature stress increased. All of the Se treatments significantly decreased the H_2_O_2_ content and protected the plant membrane (Figure 2e,f). In general, the C1 and C2 treatments produced more protective effects than the C3 treatment.

### 2.3. Antioxidant Enzyme Activities

**Figure 3:**
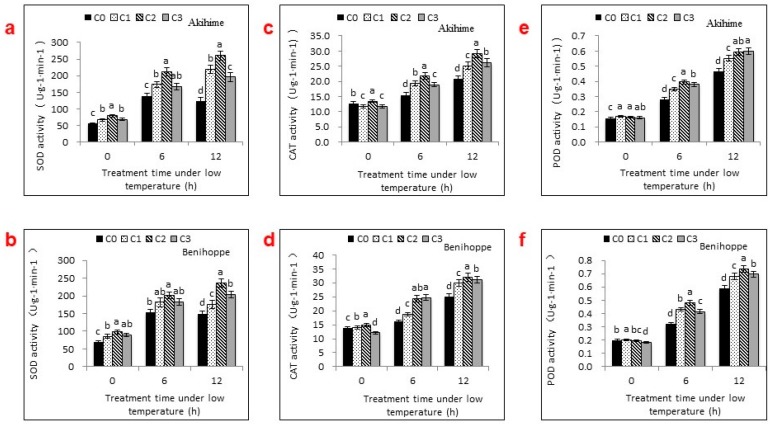
Effects of different exogenous Se treatments (C0, 0 mg·L^−1^; C1, 2.5 mg·L^−1^; C2, 5 mg·L^−1^; C3, 10 mg·L^−1^) on the activity levels of the antioxidant enzymes SOD (**a**,**b**), CAT (**c**,**d**), and POD (**e**,**f**) in the leaves of two strawberry varieties under chilling stress. Bars with different letters are significantly different at the 0.05 level (LSD test).

As shown in Figure 3, the SOD activity increased as the time under chilling stress increased from 0 to 6 h and then decreased from 6 to 12 h. Furthermore, Se applications resulted in a further significant increase in SOD activity, with greater effects observed after 6 h at 0 °C than after 12 h.

Significant effects of Se applications on strawberry seedlings under chilling stress were observed for CAT and POD (*p* < 0.05) activity levels (Figure 3). From C1 to C3, the CAT activity increased 25.16%, 40.11%, and 21.62%, respectively, compared with C0 for Akihime and it increased 16.72%, 51.00%, and 53.85% for Benihoppe, respectively, after 6 h at 0 °C. After 12 h, as the chilling stress strengthened, the effects of Se applications became more obvious. The maximum increase in POD activity was observed after the C2 treatment in both varieties. There were significant variations in the POD activity levels between Se-sprayed and unsprayed plants from 0 to 6 h and 0 to 12 h under chilling stress. The variations in CAT levels were similar.

### 2.4. APX Activity and the AsA-GSH Cycle during Chilling Stress and Recovery Growth

To obtain a better understanding of the effects of Se applications on the AsA-GSH cycle, APX activity and related low-molecular metabolism were investigated during chilling stress and recovery using Akihime because of its greater sensitivity to chilling stress and Se applications. The C2 concentration was chosen for further study because of its greater effects.

**Figure 4:**
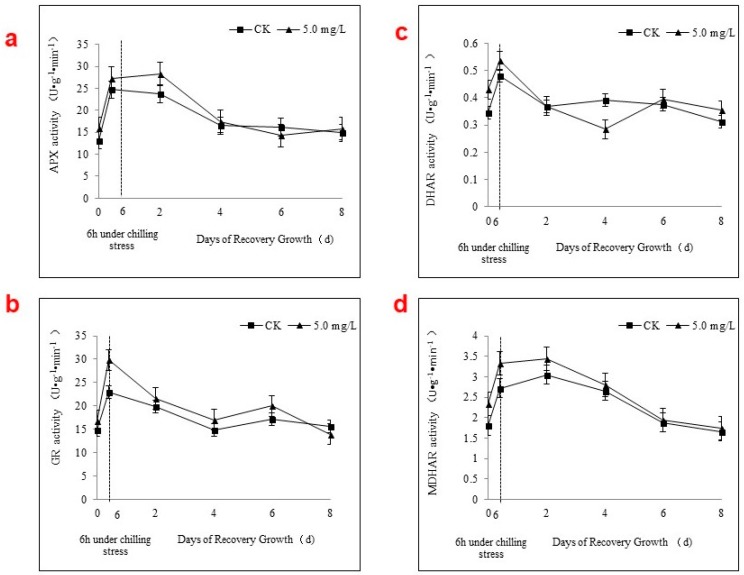
Effects of foliar spraying an Se solution (5 mg·L^−1^) on the activity levels of AsA-GSH cycle-related enzymes APX (**a**), GR (**b**), DHAR (**c**), and MDHAR (**d**) in Akihime strawberry leaves during chilling stress and recovery growth.

Chilling stress stimulated APX activity, leading to increases of 72.21% and 87.59% from 0 to 6 h for C0 and C2, respectively. Se applications significantly increased APX activity levels during chilling stress and the first initial 2 days of recovery, and then the difference gradually disappeared (Figure 4a).

After the foliar spraying of the Se solutions C0 and C2, MDHAR activity levels increased 42.80% and 50.13%, respectively, from 0 to 6 h during chilling stress (Figure 4d). During recovery growth, MDHAR activity increased significantly until day 4 but was the same as C0 by day 6. However, the foliar spraying of the Se solutions C0 and C2 resulted in the DHAR activity levels increasing 30.84% and 39.60%, respectively, from 0 to 6 h during chilling stress. However, the DHAR activity in the C2-treated leaves quickly decreased during recovery growth compared with C0 (Figure 4c). After day 4, the DHAR activity increased again in C2-treated leaves, but at 6 and 8 days, the levels were similar to those of C0. The activity level of MDHAR was approximately 6-fold that of DHAR. The activity levels of GR and MDHAR were similar.

**Figure 5:**
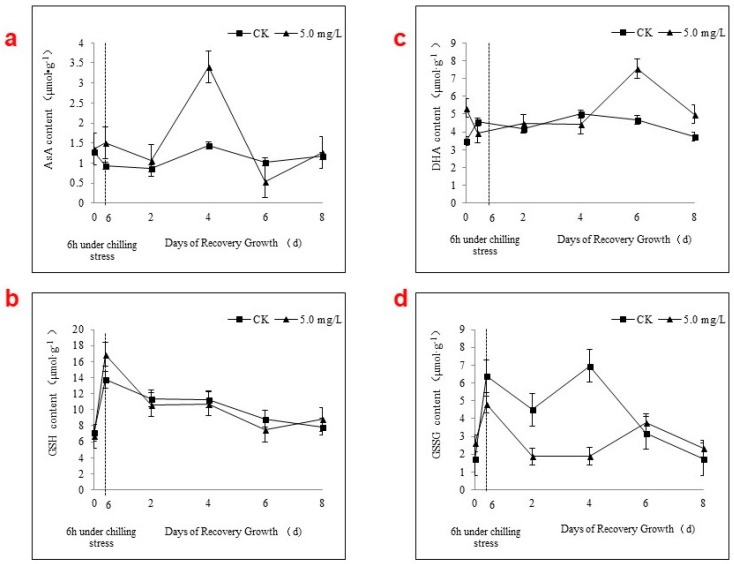
Effects of foliar spraying an Se solution (5 mg·L^−1^) on the contents of low-molecular antioxidants AsA (**a**), GSH (**b**), DHA (**c**), and GSSG (**d**) in Akihime strawberry leaves during chilling stress and recovery growth.

As shown in Figure 5a, Se applications markedly increased the AsA content of strawberry seedlings after 6 h of chilling and greatly increased the AsA content from 2 to 4 days before it quickly decreased at 6 days to a level lower than that of the control. After this time, differences between treated and untreated plants disappeared. The GSH and DHA contents changed during chilling stress and recovery growth; however, there were no clear effects of Se applications (Figure 5b,c).

Unlike the low-molecular antioxidants described above, chilling stress increased the GSSG content in the C0 seedlings without Se applications by 270.04% from 0 to 6 h. However, the C2 treatment increased the GSSG content by only 83.33% during this time (Figure 5d). During recovery growth, the GSSG contents in seedlings of both treatments decreased almost simultaneously for the first 2 days. Afterwards, the GSSG content of C0 quickly increased and then decreased more gradually than in the C2-treatment group. The C2 treatment maintained a relatively steady GSSG content from 2 to 4 days before it gradually increased and then decreased.

## 3. Discussion

Pn, Ci, and Gs are important indicators of plant growth and are affected by various environmental factors [18,19]. The Pn markedly decreased in strawberries exposed to longer periods of low temperatures. Foliar spraying with Se could significantly alleviate the damaging effects of this chilling, potentially by enhancing gas exchange during the first 6 h of chilling stress. Although the Gs under Se application treatments was still significantly greater than that of C0 after 12 h of chilling stress, the Ci increased in Se-treated plants after 0 and 6 h, indicating that the effects of Se applications on Pn increases may not arise solely from the improved Gs. Farquhar et al. [18] reported that in low-temperature environments, decreases in Pn and Gs were associated with increases in Ci, indicating that nonstomatal factors had the major limiting effects. The Se-associated increases in plant Pn values during chilling stress may not rely solely on the benefits of Gs but also on the protection of chloroplast membranes and other photosynthetic mechanisms.

The changes in chlorophyll, MDA, and H_2_O_2_ contents form a comprehensive response in plants to low-temperature stress and set a reliable standard for cold tolerance [20]. In many plants, the chlorophyll content decreases and MDA and H_2_O_2_ contents increase under low-temperature stress [21]. Here, spraying with Se solutions alleviated chlorophyll degradation in low-temperature stored seedlings and decreased the MDA and H_2_O_2_ accumulation levels, thereby maintaining the stability of the photosynthetic apparatus and membrane system in plants and increasing the chilling tolerance of strawberry seedlings. A study in which treating sorghum with an appropriate concentration (2.5–5 mg·L^−1^) of Se alleviated a reduction in the chlorophyll content, potentially by promoting the absorption of mineral elements related to chlorophyll synthesis in plants [22], supports our results. Se may participate in the synthesis of chlorophyll precursors in the form of Se-amino acids [23]. Similarly, treatment with an appropriate concentration (2.5–5 mg·L^−1^) of Se decreased the MDA contents of strawberry plants, while a higher concentration of Se (>10 mg·L^−1^) had the opposite effect. High concentrations of Se may substitute for sulfur inside the plant, participating in sulfur-related protein metabolism and disrupting protein synthesis, structure, and function [24]. The low dose of Se used in foliar spraying also decreased the H_2_O_2_ content, thereby contributing to a greater chlorophyll content and lower MDA content. H_2_O_2_, as an ROS, may play a dual role. At mild concentrations, it may act as a signal molecule involved in acclimation-related signaling, triggering tolerance to various stresses, while at high concentrations, H_2_O_2_ and other ROS may orchestrate programmed cell death. Se applications decreased H_2_O_2_ and MDA contents, as well as membrane damage, compared with unsprayed controls (Figure 4 and Figure 5). This is in accordance with the findings of Djanaguiraman et al. [5] in soybean and sorghum and Habibi [25] in sunflower.

To alleviate or prevent stress-induced oxidative injury, plants have evolved mechanisms to scavenge these toxic and reactive species through the antioxidation of enzymatic and nonenzymatic systems. SOD, CAT, and POD are three of the most important antioxidant enzymes. There are reports of varied effects of Se applications on SOD, CAT, and POD activities in different plants exposed to different stresses. Djanaguiraman et al. [5] reported that foliar spraying with appropriate concentrations of Se significantly increased SOD and CAT activities in sorghum leaves during high-temperature stress. Saidi et al. [9] reported that lower concentrations of Se down-regulated cadmium-induced increases in SOD and POD activity levels and up-regulated the decreased CAT activity level. In this study, SOD, CAT, and POD activity levels increased in strawberry leaves after Se applications during chilling stress, especially at lower Se concentrations. This was likely caused by the translation of cysteine into selenocysteine after exogenous Se applications and the replacement of sulfur by Se. This is consistent with the role of Se in introducing selenocysteine into the active site of the enzyme glutathione peroxidase (GSH-Px), thereby increasing the enzyme’s activity level [22]. Proietti et al. [8] proposed a similar mechanism. Recently, Gupta et al. [3] reported the effects of Se on sulfur-related gene transcripts. However, understanding the direct role of Se in determining SOD, CAT, and POD activity levels requires further study.

Recently, low-molecular antioxidants, including AsA, GSH, α-tocopherol, β-carotene, flavonoids, and hydroquinones, have attracted interest [26,27]. Most researchers, however, have paid greater attention to AsA and GSH because they can be rapidly regenerated enzymatically through the AsA-GSH cycle, which plays an important role in the removal of ROS [28,29]. The four enzymes, APX, GR, DHAR, and MDHAR, cooperate and complete the AsA-GSH cycle, maintaining the optimum AsA and GSH levels in the plant [30]. In this study, the activity levels of both APX and MDHAR increased during chilling stress from 0 to 6 h and remained high in the first 2 days of recovery growth. Furthermore, Se applications simultaneously and significantly increased APX and MDHAR activity levels. APX utilizes AsA as a specific electron donor to reduce H_2_O_2_ to water, with the concomitant generation of MDHA. MDHA is spontaneously converted to AsA and DHA and is also rapidly reduced to AsA by the action of DHAR, which first utilizes GSH to reduce DHA. GSH is then regenerated from GSSG by the action of GR using NADPH. Thus, MDHAR activity is essential for the reduction of MDHA to meet the demand for AsA. DHAR and GR activity levels rapidly increased during chilling stress and decreased during recovery growth before gradually returning to their original levels. That indicated that APX was able to oxidize AsA and that MDHAR was a key enzyme for the regeneration of AsA in the AsA-GSH cycle, with Se applications further enhancing this function. A previous report from Luo et al. [2] regarding the AsA-GSH cycle in strawberry leaves exposed to chilling stress supports our results. The large increase in the AsA content during recovery from 2 to 4 days may be caused by the greater MDHAR activity level in the first 2 or 3 days. Conversely, the GSSG content of the control group was significantly greater than that of the Se-treated plants, potentially because of the lower DHAR and GR activity levels in the control group. However, related reports are limited [17]. GSH-Px activity is also related to GSSG production and is closely related to Se metabolism and the AsA-GSH cycle [31]. Further studies on the impacts of Se on GSH-Px in stress-treated strawberry plants are therefore required.

There may be concerns regarding the safety of Se-treated strawberry fruit [32]. However, in China, ~72% of the counties are located in Se-deficient areas [33]. A 20–40 g·hm^−2^ sodium selenite foliar spray at the early flowering stage has been recommended to produce Se-enriched strawberry fruit, and the Se content of the first batch of strawberry fruit met the Se-enriched fruit standard provided by Shanxi Province [33,34]. However, there is currently no national or international Se-enriched food standard. In this study, the Se foliar-spray treatments of ~1.5, 3, and 6 g per hectare were much lower than the treatments recommended for Se-enriched strawberry fruit production and obviously lower than U.S. Department of Agriculture recommended dietary allowance of 50–70 μg Se d^−1^ for regular adults [35]. We confirmed that there is no risk when a Se (sodium selenite) solution is used as a plant regulator in the Se-deficient areas of China. However, it may be useful to determine the changes in the Se contents and states at different harvest stages after Se treatments.

In conclusion, foliar spraying of appropriate concentrations of Se increased the photosynthesis, antioxidant enzyme activity levels, and low-molecular antioxidant content and promoted the AsA-GSH cycle of strawberry seedlings during chilling stress. MDHAR was the key affected enzyme in the AsA-GSH cycle. These results will provide potential measures to aid strawberry farmers in coping with a chilling disaster.

## 4. Materials and Methods

### 4.1. Plant Materials and Growth Conditions

Experiments were carried out at the Agricultural Experiment Station of Zhejiang University (AES-ZJU) from October 2015 to January 2016. Plantlets of strawberry cultivars Akihime and Benihoppe were purchased from a field-cropping seedling nursery garden (Hongqun Farmer-specialized Cooperative, Jiande County, Zhejiang Province, China) located in the largest strawberry-cropping county in Zhejiang Province. The seedlings were transferred to plastic pots (top and bottom diameters of 20 cm and 15 cm, respectively; pot depth of 13 cm) with 800 g loam soil from the Huajiachi Campus’ experimental field of AES-ZJU. The soil pH was 6.8, with 25.62 g·kg^−1^ total soil organic matter content, 1.54 g·kg^−1^ total nitrogen, 44.8 mg·kg^−1^ available phosphorus, 46.8 mg·kg^−1^ available potassium, and 0.03 mg·kg^−1^ total Se. Every pot contained one four-leaf stage seedling. These seedlings were cultivated in a greenhouse at the Zijingang Campus of AES-ZJU with a 28/20 °C day/night temperature regime and natural sunlight before the chilling treatment. The plants were irrigated once every 2 to 3 days to avoid water stress. Consistent with field production conditions, 1 g compound fertilizer (20%:20%:20% N:P:K, respectively; provided by AES-ZJU) was applied to each pot every month. After a new leaf was fully spread, healthy and uniform seedlings (with 4.5 to 5 leaves) were selected for experiments.

### 4.2. Se Treatment and Chilling Stress

The strawberry seedlings grown as described in Section 2.1 were placed into two climate chambers (AGCM-113DC01, Hangzhou, China) with regulated temperatures of 25/20 °C day/night, 12-h photoperiod with a PPDF of 360 μmol·m^−2^·s^−1^ and a relative humidity (RH) of 80% ± 5% for 7 days as the pretreatment. After the pretreatment, the seedlings were foliar sprayed with Se at three concentrations (2.5, 5, and 10 mg·L^−1^, abbreviated as C1, C2, and C3, respectively) or distilled water as the control (C0). There were a total of 40 trays of plants per experiment, including three replicates of each treatment with 10 trays per replicate. Plants were arranged randomly. Plants of each treatment were sprayed with 20 mL of a sodium selenite (Na_2_SeO_3_) solution twice daily at 8 a.m. and 5 p.m. for 3 consecutive days (a total of 6 times) before chilling stress. Chilling stress was induced at 0 °C for 0, 6, or 12 h at 80% ± 5% RH, followed by 8 days of recovery growth under the pretreatment conditions. The temperature was gradually decreased from the regular regime to chilling stress over a 24-h period. From chilling stress to recovery growth, the opposite occurred. A recovery analysis was only conducted for Akihime because it was more sensitive to chilling stress. The recovery analysis was performed after 6 h of chilling stress. The first and third mature leaves of each plant in all of the treatments were taken for determinations. These were frozen in liquid nitrogen and stored at −70 °C.

### 4.3. Determination of Chlorophyll Content and Photosynthetic Parameters

To determine the chlorophyll content, 0.5 g of frozen leaf samples were homogenized with 10 mL of acetone (80%, *v*/*v*) using a precooled pestle and mortar. Then, the homogenate was centrifuged at 5000× *g* for 10 min. The absorbance levels were measured with a UV-visible spectrophotometer (Beckman, CA, USA) at 663 and 645 nm. The chlorophyll content was calculated using the equations proposed by Lichtenthaler [36]. The Pn, stomatal conductance (Gs), and intracellular CO_2_ concentration (Ci) were determined in the chilled plants using an LI-6400 portable photosynthesis equipment (LI-COR, USA). The air temperature, RH, CO_2_ concentration, and photosynthetic photon flux density (PPFD) were maintained at 25 °C, 85%, 380 µmol·mol^−1^, and 1000 µmol·m^−2^·s^−1^, respectively.

### 4.4. Determination of Malondialdehyde (MDA) and H_2_O_2_ Contents

The MDA level was assayed using the thiobarbituric acid reaction as described by Wu et al. [37]. H_2_O_2_ levels were measured by monitoring the absorbance at 410 nm of the titanium-peroxide complex following the method described by Lin et al. [38]. Briefly, 1 mL of cold acetone-extracted supernatant was added to 0.1 mL 5% titanium reagent (5% *w*/*v* Ti(SO_4_)_2_·9H_2_O) and 0.2 mL 17 M ammonia solution. The solution was centrifuged at 3000× *g* at 4 °C for 10 min and the supernatant was discarded. The pellet was dissolved in 5 mL 1 M sulfuric acid, and the absorbance was measured with a UV-visible spectrophotometer (Beckman) at 410 nm. Absorbance values were calibrated to a standard curve generated using known concentrations of H_2_O_2_ and expressed in μmol·g^−1^ fresh weight.

### 4.5. Determination of Antioxidant Enzyme Activities

Total SOD activity was determined as described by Prochazkova et al. [39]. Leaf samples of 1 g (fresh weight) were ground with a pestle in an ice-cold mortar with 8 mL of extraction buffer as described by Ali et al. [40]. The 50 mM phosphate buffer (pH 7.8) for SOD extraction contained 0.1 mM ethylenediaminetetraacetic acid (EDTA), 0.5% (*m*/*v*) polyvinylpyrrolidone, and 0.1% Triton X-100. The homogenates were filtered through four layers of gauze and then centrifuged at 12,000× *g* for 20 min at 4 °C. The supernatants were collected and used to assay antioxidant enzyme activities. The 50 mM phosphate buffer (pH 7.8) for CAT extraction contained 1% (*m*/*v*) polyvinylpyrrolidone, 0.1% TritonX-100, and 0.1 mM EDTA. Peroxidase (POD) activity was assayed according to the method of Putter [41], with some modifications. The reaction mixture consisted of 100 μL enzyme extract, 100 μL guaiacol (1.5%, *v*/*v*), 100 μL 300 mM H_2_O_2_, and 2.7 mL 25 mM potassium phosphate buffer with 2 mM EDTA (pH 7.0).

### 4.6. Determination of APX Enzyme Activity and the AsA-GSH Cycle

APX activity was measured according to the method of Nakano and Asada [42] by following the decrease in the absorbance at 290 nm. The assay mixture contained 100 μL enzyme extract, 100 μL 7.5 mM AsA, 100 μL 300 mM H_2_O_2_, and 2.7 mL 25 mM phosphate buffer (pH 7.0). Glutathione reductase (GR) activity was measured according to the method of Garcia-Limones et al. [43] by following the decrease in the absorbance at 340 nm. The reaction mixture contained 100 μL enzyme extract, 100 μL 10 mM oxidized glutathione (GSSG), 100 μL 24 mM nicotinamide adenine dinucleotide phosphate (NADPH), and 2.7 mL 25 mM phosphate buffer with 2 mM EDTA (pH 7.0). Monodehydroascorbate reductase (MDHAR) was measured according to Aravind et al. [44] by following the decrease in the absorbance at 340 nm. The reaction mixture contained 200 μL enzyme extract, 590 μL 25 mM phosphate buffer with 2 mM EDTA (pH 7.0), 100 μL 7.5 mM AsA, 100 μL 2 mM NADH, and 10 μL (2.5 units) of AsA oxidase. Dehydroascorbate reductase (DHAR) was measured according to the method of Dalton et al. [45] by following the decrease in the absorbance at 265 nm. The assay solution contained 50 μL enzyme extract, 750 μL 25 mM phosphate buffer with 2 mM EDTA (pH 7.0), 100 μL 20 mM GSH, and 100 μL 24 mM NADPH.

Fresh leaf samples (0.2 g) were ground in 5 mL 10% (*w*/*v*) trichloroacetic acid at 2 °C and centrifuged at 15,000× *g* for 10 min. Then, the supernatant was used to determine the contents of AsA, total ascorbate, dehydroascorbic acid (DHA), GSSG, total glutathione, and GSH. The AsA and total ascorbate contents were measured according to the method of Hodges et al. [46]. Total ascorbate was measured after the sample was incubated in dithiothreitol for 15 min. The DHA content was estimated from the difference between total ascorbate and AsA. A standard curve prepared using AsA and DHA was used to calculate the amounts of total ascorbate, AsA, and DHA. GSSG and total glutathione contents were measured according to the method of Griffith [47]. The GSSG content was determined after the removal of GSH by 2-vinylpyridine derivatization. The GSH content was then estimated from the difference between total glutathione and GSSG. A standard curve prepared using GSH and GSSG was used to calculate the amounts of total glutathione, GSH, and GSSG.

### 4.7. Statistical Analyses

The statistical analysis was performed using a one-way analysis of variance (ANOVA). Comparisons between the treatment means were performed using a least significant difference test at the *p* ≤ 0.05 level.

## Abbreviations

AES-ZJU	Agricultural Experiment Station of Zhejiang University
AsA	Ascorbic acid
APX	Ascorbate peroxidase
CAT	Catalase
DHAR	Dehydroascorbate reductase
GR	Glutathione reductase
GSH	Glutathione
GSSG	Oxidized glutathione
H_2_O_2_	Hydrogen peroxide
MDA	Malondialdehyde
MDHAR	Monodehydroascorbate reductase
POD	Peroxide enzyme
ROS	Reactive oxygen species
SOD	Superoxide dismutase
